# Genome-wide interval mapping using SNPs identifies new QTL for growth, body composition and several physiological variables in an F_2_ intercross between fat and lean chicken lines

**DOI:** 10.1186/1297-9686-45-36

**Published:** 2013-09-30

**Authors:** Olivier Demeure, Michel J Duclos, Nicola Bacciu, Guillaume Le Mignon, Olivier Filangi, Frédérique Pitel, Anne Boland, Sandrine Lagarrigue, Larry A Cogburn, Jean Simon, Pascale Le Roy, Elisabeth Le Bihan-Duval

**Affiliations:** 1 INRA, UMR1348 PEGASE, 35042 Rennes, France; 2 Agrocampus Ouest, UMR1348 PEGASE, 35042 Rennes, France; 3 INRA, UR83 Recherches Avicoles, 37380 Nouzilly, France; 4 INRA, UMR444 Génétique Cellulaire, 31326 Castanet-Tolosan, France; 5 CEA, IG, Centre National de Génotypage, 2 rue Gaston-Crémieux, CP 5721, 91057 Evry, France; 6 Department of Animal and Food Sciences, University of Delaware, Newark, DE 19717, USA

## Abstract

**Background:**

For decades, genetic improvement based on measuring growth and body composition traits has been successfully applied in the production of meat-type chickens. However, this conventional approach is hindered by antagonistic genetic correlations between some traits and the high cost of measuring body composition traits. Marker-assisted selection should overcome these problems by selecting loci that have effects on either one trait only or on more than one trait but with a favorable genetic correlation. In the present study, identification of such loci was done by genotyping an F_2_ intercross between fat and lean lines divergently selected for abdominal fatness genotyped with a medium-density genetic map (120 microsatellites and 1302 single nucleotide polymorphisms). Genome scan linkage analyses were performed for growth (body weight at 1, 3, 5, and 7 weeks, and shank length and diameter at 9 weeks), body composition at 9 weeks (abdominal fat weight and percentage, breast muscle weight and percentage, and thigh weight and percentage), and for several physiological measurements at 7 weeks in the fasting state, i.e. body temperature and plasma levels of IGF-I, NEFA and glucose. Interval mapping analyses were performed with the QTLMap software, including single-trait analyses with single and multiple QTL on the same chromosome.

**Results:**

Sixty-seven QTL were detected, most of which had never been described before. Of these 67 QTL, 47 were detected by single-QTL analyses and 20 by multiple-QTL analyses, which underlines the importance of using different statistical models. Close analysis of the genes located in the defined intervals identified several relevant functional candidates, such as *ACACA* for abdominal fatness, *GHSR* and *GAS1* for breast muscle weight, *DCRX* and *ASPSCR1* for plasma glucose content, and *ChEBP* for shank diameter.

**Conclusions:**

The medium-density genetic map enabled us to genotype new regions of the chicken genome (including micro-chromosomes) that influenced the traits investigated. With this marker density, confidence intervals were sufficiently small (14 cM on average) to search for candidate genes. Altogether, this new information provides a valuable starting point for the identification of causative genes responsible for important QTL controlling growth, body composition and metabolic traits in the broiler chicken.

## Background

For decades, major genetic improvements in growth and body composition of broiler chickens have been achieved by standard selection based on pedigree and phenotypic information. For body weight (BW), which has a moderate heritability (~0.35), standard selection has been very efficient because measurement of this phenotype is easy and inexpensive, even for large populations. For body composition, such as breast muscle (BMW) or abdominal fat (AFW) weight, despite a greater heritability (~0.50), genetic progress has been slower for two reasons: (1) intensity of selection has been lower because of the difficulty and cost to measure these phenotypes, and (2) genetic evaluations have been less accurate because evaluation of candidates is based on information from relatives only. In addition, over the years, the genetic gain for growth has been accompanied by a large increase in carcass fatness [1], which itself has a negative impact on both feed efficiency and the environment via a higher excretion of nitrogen and phosphate [2-4]. Genetic selection against fatness is hindered by the high positive phenotypic correlation between body weight and fat content (*r* = 0.74) [5].

For many traits, and more specifically for those under lower selection pressure, genetic variability must still be high within and across chicken populations. This variability has been studied in many different breeds or selected lines for 20 years through quantitative trait locus (QTL) detection (for a review, [6]). However, information provided by traditional microsatellite-based QTL analyses cannot be used in selection programs because of very large confidence intervals on QTL location. More recently, whole-genome sequencing of multiple chicken breeds has led to the identification of thousands of single nucleotide polymorphisms (SNPs), providing very high-density genetic maps compared to the previously low-density microsatellite marker maps [7].

Despite the poor correlation between growth and body composition performances, it has been possible to divergently select experimental fat (FL) or lean (LL) broiler chicken lines that have different body compositions but quite similar body weights [5]. A difference in energy metabolism between such divergent lines has been highlighted [8]. FL chickens generally exhibit lower plasma glucose levels than LL chickens, unlike what is typically observed in obese mammals. Regardless of their nutritional status (fed or fasted), total plasma lipid and lipoprotein levels are higher in FL than in LL chickens, suggesting a higher rate of hepatic lipogenesis in FL chickens. The plasma level of non-esterified fatty acids (NEFA) is also higher in FL chickens, possibly because they have more adipocytes in their abdominal fat depot [9]. Differences have also been reported for several hormones, including IGF1, which has a higher level in plasma in FL than in LL chickens, regardless of the nutritional state [8]. Previous studies on these lines have determined that alleles of QTL that affect growth and body composition are not fixed in the FL or LL lines [10,11].

Using SNPs, the present study aimed at detecting new QTL that affect 17 traits related to growth, body composition and several relevant physiological variables in a FL × LL F_2_ population. A medium-density genetic map (1422 markers) was used, which is much lower than what would be obtained with 60 K SNP arrays, and does not allow for linkage disequilibrium analyses, but it is dense enough for fine mapping of QTL using linkage analyses, and the selected markers cover the whole sequenced genome. Different interval mapping strategies were applied using QTLMap software: single-trait analyses with single and multiple QTL on the same chromosome [12,13].

## Methods

### Animals

A three-generation design was used at the INRA PEAT experimental farm (Nouzilly, Indre-et-Loire) by inter-crossing two experimental meat-type chicken lines, i.e. the fat (FL) and lean (LL) lines, that had been divergently selected for seven generations using the abdominal fat weight/animal weight ratio as an index of fattening, while reaching quite similar live body weights at 9 weeks [5]. After selection, the two lines were maintained by limiting inbreeding. In the F_0_ generation, five FL males were mated to 13 unrelated LL females and four LL males were mated with eight unrelated FL females to generate the F_1_ generation. Five F_1_ males (three from an FL × LL cross and two from an LL × FL cross) were each mated to nine or 10 unrelated F_1_ dams to produce 579 F_2_ progeny that were reared in five successive hatching groups. Blood was collected from all chickens for DNA analyses. The F2 chickens were raised under similar conditions (one floor pen per hatch) and fed *ad libitum* using conventional feed: a starter ration from 0 to 3 weeks of age (metabolizable energy: 3050 kcal/kg, crude protein: 220 g/kg, lysine: 12 g/kg, methionine + cystine: 8.5 g/kg, tryptophan: 2.5 g/kg, threonine: 8.3 g/kg, calcium: 11 g/kg, available phosphorus: 4.2 g/kg), and a grower ration from 4 to 9 weeks of age (metabolizable energy: 3100 kcal/kg, crude protein: 200 g/kg, lysine: 11.3 g/kg, methionine + cystine: 8.22 g/kg, tryptophan: 2.27 g/kg, threonine: 7.38 g/kg, calcium: 8.96 g/kg, available phosphorus: 3.8 g/kg). To synchronize biological and metabolic rhythms, the chickens were maintained under a 14-hour light/10-hour darkness cycle (14L10D), with lights on from 9 am to 11 pm. Chickens were slaughtered at nine weeks of age and carcasses were eviscerated and stored at 4°C for 20 hours before dissection. All procedures were conducted under Licence No. 37–123 from the Veterinary Services, Indre-et-Loire, France and in accordance with guidelines for Care and Use of Animals in Agricultural Research and Teaching (French Agricultural Agency and Scientific Research Agency).

### Phenotypic measurements

Body weight was measured at 1, 3, 5, 7 and 9 weeks of age, i.e. BW1, BW3, BW5, BW7, and BW9, respectively. Body composition traits measured at 9 weeks included breast muscle percentage (BMP), pectoralis minor (BMWmin) and pectoralis major (BMWmaj) weights, abdominal fat weight (AFW) and percentage (AFP), thigh weight (ThW) and percentage (ThP), and shank diameter (ShD) and length (ShL). Weights were expressed in grams. Lengths and diameters were expressed in millimeters. For physiological measurements, blood samples were collected at 7 weeks of age from the wing vein with syringes containing EDTA as anticoagulant, after an overnight fast. To minimize stress, chickens were placed in crates at 4 pm on the day prior to sample collection and lights were turned off. On the day of sampling, chickens were maintained in darkness until body temperature (*T*_b_) was measured, and then blood samples were collected and kept on ice. *T*_b_ (in °C) was measured in the cloaca with an electronic thermometer (Testo 110, Testo, Forbach, France) while handling and restraining the chickens gently.

The level of fasting plasma glucose (glucose) was measured by the glucose oxidase method (Glucose Beckman Analyzer 2, Beckman, Palo Alto, CA) and expressed in mg/dL. The level of non-esterified fatty acids (NEFA) level was determined with an enzymatic colorimetric kit (Wako, Chemicals, Neuss, Germany) and expressed in mEq/L. Fasting plasma IGF-I levels were measured by radioimmunoassay, as previously described by Enright *et al*. [14] and expressed and analyzed as pg/10 μl of plasma diluted at a ratio of 1/7.

### Marker selection and genotyping

The five F_1_ sires were genotyped for a set of 9216 SNPs covering the 28 first autosomes and the GGAZ (*Gallus gallus* chromosome Z). A subset of 1536 SNPs was selected using MarkerSet software [15] based on SNP location and heterozygozity in the F_1_ population to maximize both genome coverage and marker informativity. All F_2_ animals were genotyped for these 1536 SNPs and for 120 microsatellites that were used in a previous analysis [11]. SNP genotyping was performed at the National Genotyping Center (CNG, Evry, France) using Illumina GoldenGate technology (Illumina, San Diego, CA, USA). For microsatellite genotyping, two to 10 markers were combined for multiplex PCR amplification based on size and amplification conditions and analyzed on an automated sequencer (ABI 3700, PE Applied Biosystems, Foster City, CA). The marker length and genotype of the animals were determined using GeneScan and Genotyper software (Applied Biosystems, Version 3.7). Mendelian errors were corrected using the MendelSoft software [16].

### QTL mapping

When needed, the GLM procedure of SAS was used to adjust traits for sex and hatch groups (fixed effects) and BW9 (covariate). BW9 was not used as covariate for BW1, BW3, BW5, BW7, ThP and IGF-I. Correlations between adjusted traits were then calculated using the "cov2cor" function in R [17]. The significance level for the bilateral correlation test for more than 500 values and *P* < 0.01 was 0.115, using the Bravais-Pearson *r* table.

Heritabilities and genetic correlations of the measured traits were estimated by the REML method with the VCE6 software [18]. The model included the fixed effects of hatch (*N* = 5) and sex (*N* = 2), the animal genetic effect (*N* = 623), as well as BW9 as a covariate (except for BW1, BW3, BW5, BW7, ThP and IGF-I, as previously mentioned). For body weight traits, a maternal random effect (*N* = 47) was added.

Genetic locations of the markers were extracted from the international consensus map [19]. The location of markers that were not included in the consensus map was extrapolated from that of flanking markers. Briefly, considering marker *m* with flanking markers *a* and *b*, the local ratio of genetic (cM = centimorgans) to physical (Phy) distance [ratio = (cM_a_–cM_b_)/(Phy_a_–Phy_b_)] was used to calculate the genetic location of the marker based on its physical distance from the previous marker cM_m_ = cM_a_ + [(Phy_m_–Phy_a_) × ratio]. A total of 234 markers had no reliable physical location or presented technical problems (low call rate and/or high Mendelian errors), so only 1422 of the 1656 markers (1536 SNPs and 120 microsatellites) were selected for the analysis.

A linkage QTL interval mapping analysis was performed using the QTLMap software (http://www.inra.fr/qtlmap), which was developed for outbred experimental populations [12,13], taking into account the familial structure of the population (five half-sib sire families and heteroscedastic model). Fixed and covariate effects were taken into account as previously, i.e. when they were significant at the 5% level in the GLM, and were estimated jointly with the QTL parameters. No assumptions about fixation of alleles in the founder lines and the number of the alleles segregating at QTL were made (i.e. a separate QTL effect was estimated for each sire). This was particularly important with this population, since it has been previously demonstrated that most of the QTL detected, including those that affect abdominal fatness, a highly selected trait, were not fixed in the founder lines [10,11]. A fast algorithm was used to estimate transmission probabilities at each location of a chromosome based on the SNP genotypes [20], which made it possible to use a large set of markers. A specific GPU accelerated version of QTLMap was used to reduce computation time [21]. The presence of QTL was assessed using a likelihood ratio test (LRT) under the hypothesis of one versus no QTL linked to a given set of markers [12]. The QTLMap software was also used to test more complex hypotheses, such as whether two linked QTL influence the same trait [22]. Two approaches were used for multiple-QTL analysis. When one QTL was identified in a chromosome, the hypothesis of one QTL in the chromosome (H1) was compared to the hypothesis of two QTL in the chromosome (H2). This is of particular interest to test whether a QTL detected in a single-QTL linkage analysis was a ghost, or whether another QTL might be segregating elsewhere on the chromosome. When no QTL was detected for a chromosome by trait combination, the hypothesis of no QTL on the chromosome (H0) was compared to the H2 hypothesis to test for segregation of two antagonistic QTL. In all cases, the two QTL locations under H2 were estimated considering all possible combinations of locations, using a two-dimensional grid. Since the heterogametic sex in chicken is the male, it was possible to perform within-sire family analyses on GGAZ. Confidence intervals of QTL positions were estimated by the drop-off method [23].

For all QTL analyses, significance thresholds were determined by simulating phenotypes under the null hypothesis of the test to obtain an empirical distribution of the likelihood ratio test (LRT) in accordance with the pedigree and marker information. Thus, for the no-versus-one QTL test, phenotypes were simulated under H0, assuming a polygenic model with the trait heritability detailed in Table 1. For the two-versus-one QTL test, performances were simulated under H1. The most likely location and effect estimated during the no-versus-one QTL test were used to determine the QTL effect on phenotype. The QTL effect used in these simulations was the average of the effects of all heterozygous sires. One thousand and 10 000 (for multiple-QTL and single-QTL analyses, respectively) simulations were performed for each trait by chromosome combinations, and quantiles of maximum LRT were calculated according to Harrel and Davis [24].

**Table 1:** **Descriptive statistics and heritabilities (*h***^**2**^**) of recorded traits**

**Trait**	**Males**			**Females**			**All**		
	***N***	**Mean**	**STD**	***N***	**Mean**	**STD**	**Mean**	**STD**	***h***^**2**^
BW1	274	97.67	14.18	290	94.15	13.39	95.86	13.87	0.32 ± 0.03
BW3	271	460.7	54.29	296	413.36	50.08	435.99	57.21	0.36 ± 0.03
BW5	272	1069.38	109.79	296	907.29	99.43	984.91	132.18	0.41 ± 0.04
BW7	266	1690.7	177.42	296	1380.42	139.27	1527.28	221.61	0.32 ± 0.02
BW9	276	2507.05	262.84	298	1979.05	201.42	2232.93	351.99	0.22 ± 0.02
AFW	273	71.93	21.73	292	73.97	22.17	72.98	21.96	0.61 ± 0.15
AFP	274	2.85	0.76	292	3.7	0.93	3.29	0.95	0.63 ± 0.05
BMWmaj	273	110.62	13.58	292	89.15	10.13	99.53	16.03	0.47 ± 0.05
BMWmin	274	37.87	4.57	292	31.37	3.36	34.52	5.15	0.40 ± 0.06
BMP	273	11.82	0.86	290	12.17	0.94	12.02	0.91	0.59 ± 0.05
ThW	273	301.92	36.23	291	229.38	25.73	264.49	47.87	0.33 ± 0.03
ThP	278	24.04	1.19	291	23.13	1.08	23.58	1.22	0.37 ± 0.04
ShD	268	12.59	0.79	297	10.92	0.59	11.72	1.08	0.45 ± 0.03
ShL	275	123.38	5.92	296	108.37	4.61	115.60	9.17	0.44 ± 0.03
*T*_b_	272	41.85	0.27	293	41.88	0.25	41.87	0.26	0.31 ± 0.08
Glucose	274	196.7	11.78	295	196.28	11.04	196.48	11.40	0.19 ± 0.08
NEFA	261	0.488	0.098	269	0.496	0.099	0.49	0.10	0.16 ± 0.06
IGF-I	272	38.01	8.36	295	38.4	8.93	38.22	8.65	0.38 ± 0.11

*BW1, 3, 5, 7, 9* body weight at age 1, 3, 5, 7 and 9 weeks (g), *AFW and AFP* abdominal fat weight (g) and percentage (%), *BMWmin and BMWmaj* pectoralis minor and major weights (g), *BMP* breast muscle percentage (%), *ThW and ThP* thigh weight (g) and percentage (%), *ShD and ShL* shank diameter and length (mm), *T*_b_, body temperature (°C), *Glucose* fasting plasma glucose level (mg/dL), *IFG-I and NEFAs* fasting plasma IGF-I (pg/10 μl of plasma diluted at 1/7) and non-esterified fatty acid levels (mEq/L); STD are standard deviation of the traits, expressed in trait units.

At the position with the highest LRT, the substitution effect of QTL alleles was estimated in each sire family and significance of the effect (difference from 0) was tested by a *t*-test, using the within-family residual standard error, i.e. the intra QTL genotype distribution of the trait adjusted for the other effects in the model (fixed, covariate and polygenic). The additive value of the QTL effect was assessed as the average of significant substitution effects in the sires (*P* < 0.05).

## Results and discussion

### Phenotypic and genetic correlations between residuals

A description of traits, along with Pearson correlation coefficients and estimated genetic correlations between adjusted traits are in Tables 1 and 2, respectively. No correlations were presented for BW9 since it was used as covariate to adjust the other traits. Significant (*P* < 0.01) phenotypic correlations were either higher than 0.115 or lower than -0.115. As expected, correlations between BW measured at different ages were positive, and ranged from 0.21 (for BW1 and BW7) to 0.93 (for BW5 and BW7). Abdominal fatness, expressed either as total weight (AFW) or percentage of BW (AFP), was negatively phenotypically correlated with several composition traits, i.e. BMP (-0.26), BMWmaj (-0.3), ThW (-0.14), ThP (-0.14), ShL (-0.24) and ShD (-0.38) and the metabolite glucose (-0.2). Glucose was also negatively correlated with NEFA (-0.20) but positively correlated with body temperature (0.28). No significant correlations were observed for AFW and AFP with BMWmin. ThW and ThP were positively correlated with BMP (0.21) and BMWmaj (0.26) but had no significant correlation with BMWmin (-0.01). ShL was positively correlated with BW3 and BW5, while ShD was negatively correlated with BW1 (-0.17) and BW3 (-0.13). Positive correlations were found between shank and thigh traits (ShL, ShD, ThW and ThP). Finally, white muscle traits (BMP, BMWmaj and BMWmin) were positively correlated with *T*_b_ (0.19) and negatively correlated with plasma IGF-I levels (-0.13).

**Table 2:** Pearson correlation coefficients (phenotypic, above the diagonal), estimated genetic correlations (with their standard errors, below the diagonal) and heritabilities (diagonal) for the measured traits

	**BW1**	**BW3**	**BW5**	**BW7**	**AFW**	**AFP**	**BMW maj**	**BMW min**	**BMP**	**ThW**	**ThP**	**ShD**	**ShL**	***T***_**b**_	**Gluc**	**NEFA**	**IGF-I**
BW1	0.32	**0.63**	**0.30**	**0.21**	0.05	0.05	0.01	**0.16**	0.06	-0.03	-0.01	**-0.17**	0.08	-0.07	-0.06	0.00	-0.11
BW3	**0.87**	0.36	**0.54**	**0.37**	0.08	0.08	-0.04	0.06	-0.02	-0.04	-0.01	**-0.13**	**0.21**	-0.06	-0.05	0.08	-0.05
	(0.05)																
BW5	**0.74**	**0.94**	0.41	**0.93**	0.04	0.05	-0.03	0.05	-0.04	-0.09	-0.06	-0.08	**0.12**	-0.04	-0.08	0.05	-0.08
	(0.11)	(0.04)															
BW7	**0.64**	**0.72**	**0.72**	0.32	0.01	0.02	-0.02	0.01	-0.05	-0.07	-0.03	-0.04	0.08	-0.04	-0.06	0.03	-0.05
	(0.18)	(0.13)	(0.13)														
AFW	0.09	0.17	0.34	0.27	0.61	**0.99**	**-0.30**	-0.05	**-0.26**	**-0.15**	**-0.14**	**-0.38**	**-0.24**	-0.09	**-0.20**	0.11	0.04
	(0.20)	(0.18)	(0.19)	(0.24)													
AFP	0.14	0.21	**0.38**	0.33	**1.0**	0.63	**-0.30**	-0.05	**-0.26**	**-0.14**	**-0.13**	**-0.37**	**-0.24**	-0.09	**-0.20**	0.11	0.04
	(0.20)	(0.19)	(0.18)	(0.23)	(0.00)												
BMW maj	-0.30	**-0.40**	-0.31	-0.07	-0.22	-0.25	0.47	**0.57**	**0.96**	**0.26**	**0.26**	0.05	-0.08	**0.17**	0.08	-0.07	-0.11
	(0.19)	(0.17)	(0.20)	(0.26)	(0.19)	(0.19)											
BMW min	-0.27	-0.24	-0.18	-0.18	0.17	0.14	**0.66**	0.40	**0.75**	-0.01	-0.01	-0.08	0.03	**0.14**	-0.02	0.01	**-0.15**
	(0.21)	(0.21)	(0.23)	(0.30)	(0.21)	(0.21)	(0.12)										
BMP	-0.31	**-0.39**	-0.33	-0.15	-0.16	-0.18	**0.97**	**0.81**	0.59	**0.21**	**0.21**	0.04	-0.03	**0.19**	0.07	-0.05	**-0.13**
	(0.19)	(0.18)	(0.20)	(0.26)	(0.19)	(0.18)	(0.01)	(0.08)									
ThW	0.11	0.03	0.22	**0.54**	0.02	0.05	0.29	0.20	0.26	0.33	**0.98**	**0.12**	**0.25**	0.05	0.11	-0.05	0.06
	(0.22)	(0.22)	(0.22)	(0.27)	(0.22)	(0.22)	(0.21)	(0.22)	(0.19)								
ThP	0.12	0.08	0.29	**0.61**	0.06	0.10	0.24	0.20	0.22	**1.00**	0.37	**0.13**	**0.28**	0.05	0.09	-0.04	0.05
	(0.22)	(0.22)	(0.21)	(0.26)	(0.21)	(0.15)	(0.21)	(0.22)	(0.20)	(0.00)							
ShD	-0.19	-0.21	-0.18	0.09	**-0.60**	**-0.63**	**0.49**	0.17	**0.43**	0.12	0.09	0.45	0.07	0.05	**0.13**	-0.07	0.09
	(0.20)	(0.19)	(0.21)	(0.27)	(0.13)	(0.13)	(0.17)	(0.21)	(0.17)	(0.21)	(0.22)						
ShL	**0.39**	**0.53**	**0.49**	0.26	-0.09	-0.07	**-0.49**	-0.10	**-0.43**	**0.38**	0.34	-0.22	0.44	-0.04	-0.05	0.03	-0.01
	(0.18)	(0.16)	(0.17)	(0.26)	(0.18)	(0.19)	(0.17)	(0.22)	(0.18)	(0.19)	(0.20)	(0.20)					
*T*_b_	-0.09	-0.09	-0.07	0.11	**-0.58**	**-0.57**	0.33	0.10	0.37	-0.06	0.03	0.17	-0.37	0.31	**0.28**	0.01	0.03
	(0.24)	(0.23)	(0.25)	(0.33)	(0.18)	(0.17)	(0.21)	(0.25)	(0.21)	(0.24)	(0.24)	(0.22)	(0.22)				
Gluc	0.34	0.30	0.42	0.42	**-0.66**	**-0.66**	0.20	-0.22	0.13	0.04	0.03	**0.45**	-0.13	0.40	0.19	**-0.20**	0.01
	(0.29)	(0.29)	(0.29)	(0.40)	(0.18)	(0.17)	(0.28)	(0.28)	(0.30)	(0.29)	(0.28)	(0.19)	(0.29)	(0.28)			
NEFA	0.30	0.76	**0.83**	**0.73**	NE	NE	NE	0.53	-0.31	-0.04	-0.02	-0.36	NE	-0.16	0.14	0.16	0.06
	(0.36)	(0.50)	(0.21)	(0.24)				(0.37)	(0.42)	(0.33)	(0.32)	(0.34)		(0.36)	(0.47)		
IGF-I	-0.13	0.01	0.28	0.39	**-0.47**	**-0.44**	-0.05	-0.06	-0.06	0.35	0.35	0.35	**0.44**	0.39	0.29	**0.67**	0.38
	(0.23)	(0.23)	(0.21)	(0.26)	(0.21)	(0.21)	(0.23)	(0.26)	(0.23)	(0.21)	(0.22)	(0.22)	(0.21)	(0.23)	(0.26)	(0.26)	

Traits are described in Table 1; significant phenotypic (*P* < 0.01) and genetic correlations (*P* < 0.05) are in bold; *NE* could not be estimated.

Several phenotypic correlations were confirmed at the genetic level: large positive genetic correlations (ranging from 0.64 to 0.94) were found between BW at the different ages, moderate positive correlations of ShL with BW at 1 to 5 weeks of age (0.39 to 0.49), and marked negative correlations of abdominal fatness (expressed as weight or percentage) with ShD (-0.62) and plasma glucose (-0.66) (Table 2). Significant negative genetic correlations were also found for AFW and AFP with *T*_b_ (-0.58) and with IGF1 (-0.46). BW7 was positively correlated with thigh traits (0.58), while breast muscle development (BMWmaj and BMP) was positively correlated with ShD (0.46) but negatively with ShL (-0.46). Genetic correlations of NEFA with BW at 5 or 7 weeks of age and with IGF1 were positive and high (ranging from 0.67 to 0.83) but could not be estimated with abdominal fat and breast muscle traits (AFW, AFP and BMWmaj) because of lack of convergence (most probably because of the rather low heritability of NEFA).

### Genetic map

The distribution of the 1422 markers that were used is detailed in Table 3. The first 28 autosomes and GGAZ were covered by at least 17 genetic markers, except for GGA16, 25 and 27 (3, 5 and 13 markers, respectively). Chromosome GGA16 contains the major histocompatibility complex (*MHC*) and many duplications. Since this chromosome is not well covered by genome sequencing, only a few genetic markers are available, all located in an interval of less than 100 kb. Chromosomes 25 and 27 were poorly represented in the initial 9216 SNP set, and most of them were not informative in our population. The average marker interval was 2.1 cM. Since MarkerSet uses SNP locations and heterozygozity information in the F_1_ population to maximize genome coverage and marker informativity, the average marker interval was very homogeneous between chromosomes, except again for GGA16 (0 cM) and GGA25 (7.7 cM). This average marker interval was much lower than that obtained when using only microsatellite markers, i.e. 18.7 cM (data not shown); furthermore, it covers almost all chromosomes. The experimental design used for this study was based on large sire families, optimized for linkage analysis. Recent studies in layers have shown that for association analyses, the maximal distance between markers should not exceed 100 kb, and concluded that the Illumina 60 K SNP chip is appropriate for such studies [25]. Considering that the present study had one marker every 2.1 cM (or about 700 kb), using association analysis approaches would not be appropriate.

**Table 3:** Distribution of genetic markers across the chicken genome used in the analyses

**GGA**	**Number of markers**	**SNP**	**Microsatellites**	**Total length (cM)**	**Average marker interval (cM)**
1	222	202	20	445.4	2.0
2	167	151	16	312.4	1.9
3	126	113	13	268.7	2.1
4	90	82	8	202.4	2.2
5	84	75	9	152.4	1.8
6	39	37	2	110.4	2.8
7	40	33	7	113.1	2.8
8	32	28	4	90.7	2.8
9	56	52	4	88.4	1.6
10	47	44	3	82.4	1.8
11	52	47	5	69.2	1.3
12	43	41	2	73.9	1.7
13	43	39	4	57.8	1.3
14	41	37	4	67.5	1.6
15	32	29	3	55.2	1.7
16	3	3	0	0	0
17	25	25	0	51.2	2.0
18	24	22	2	50.0	2.1
19	20	20	0	52.6	2.6
20	31	27	4	51.9	1.7
21	19	19	0	45.9	2.4
22	17	17	0	58.1	3.4
23	19	19	0	45.2	2.4
24	19	19	0	43.9	2.3
25	5	5	0	38.4	7.7
26	22	17	5	45.9	2.1
27	13	11	2	52.6	4.0
28	17	14	3	51.1	3.0
Z	74	74	0	244.1	3.3
Total	1422	1302	120	3021	

*GGA Gallus gallus* chromosome.

### Single-QTL analyses

Single-QTL analyses led to the identification of 47 QTL that were located on 19 chromosomes and affected all 17 phenotyped traits, except BW1 and BW3 (Table 4). A few QTL had both a high chromosome-wide significance (*P* < 0.001) and a high genome-wide significance (*P* < 0.05). These QTL affected AFW and AFP (GGA19), BMWmin (GGA2 and GGAZ), BMP (GGA9), ShD (GGA19), IGF-I (GGA1), *T*_b_ (GGA5) and glucose (GGA18). Other QTL were also highly significant but only at the chromosome-wide level and affected AFP (GGA27, *P* < 0.01), ThP (GGA8, *P* < 0.01), and *T*_b_ (GGA11, *P* < 0.01). All other identified QTL had a chromosome-wide significance of *P* < 0.05. QTL allelic substitution effects ranged from 0.42 (BMWmaj on GGA4, BMWmin on GGA9 and glucose on GGA7) to 1.03 (BMWmin on GGA7) residual standard deviations with a mean of 0.55 standard deviations. Chromosome GGA27 was the best represented, with five identified QTL, followed by GGA3, 4, 7, 19 and 20, which each carried four QTL. The average confidence interval (CI) was equal to 14 cM, with large differences depending on the region (from 2 cM to 36 cM), mainly due to differences in marker density and informativity. However, the average CI was much smaller compared to the average CI of 32 cM observed for the same population in a study on abdominal fatness and breast muscle weight when using only microsatellites [11].

**Table 4:** Results of single-QTL analysis

**Trait**	**GGA**	**Loc (cM)**	**CI (cM)**	**Nearest markers**	**SL**	**QTL effect**	**HS**	**Ref**
ShD	1	80.5	76 - 85	rs14803813 - rs14805816	*	0.59 ± 0.17	3	-
IGF-I	1	149.5	141 - 153	rs13870138 - RBL3072	***, $	0.55 ± 0.10	4	[26,27]
ThP	1	159.5	150 - 165	rs14831825 - rs14833110	*	0.52 ± 0.11	3	-
BMW min	2	234	232 - 240	rs14231442 - rs14231751	***, $	0.69 ± 0.54	2	-
*T*_b_	2	263	256 - 267	rs13730256 - rs14246372	*	0.62 ± 0.46	2	-
BW7	3	97	92 - 100	rs14334468 - rs15316026	*	0.62 ± 0.18	2	-
ShL	3	174	163 - 180	rs14378036 - rs15394046	*	0.44 ± 0.15	4	-
Glucose	3	189	184 - 194	rs15406274 - rs14385386	*	0.47 ± 0.19	4	-
BW5	3	250	247 - 257	rs15452125 - rs14410153	*	0.65 ± 0.26	3	[28]
BMW maj	4	22	16 - 30	rs14421644 - rs14423272	*	0.42 ± 0.16	4	-
BMP	4	22	16 - 28	rs14421644 - rs14423272	*	0.49 ± 0.16	3	-
BW7	4	159	145 - 166	rs14707369 - rs14492188	*	0.60 ± 0.11	2	[29]
BW5	4	160	149 - 166	rs14492188 - rs13664708	*	0.57 ± 0.20	2	[30]
*T*_b_	5	119.9	119 - 121	rs15730058 - rs15731150	***, $	0.75 ± 0.52	2	-
ThW	6	79	71 - 82	rs15806906 - rs14588414	*	0.45 ± 0.27	3	-
ThP	6	79	72 - 82	rs15806906 - rs14588414	*	0.57 ± 0.23	2	-
Glucose	7	4	0 - 13	rs15824390 - rs13739121	*	0.42 ± 0.09	4	-
BMW min	7	34	31 - 56	rs14605238 - rs14605963	*	1.03	1	[31]
BMW maj	7	39	30 - 66	rs14606550 - rs15844013	*	0.49 ± 0.27	3	[31]
BMP	7	39	31 - 58	rs14606550 - rs15844013	*	0.49 ± 0.35	3	-
ThW	8	5.9	0 - 13	RBL4827 - rs14635367	*	0.50 ± 0.19	3	-
ThP	8	62.9	56 - 77	rs15925157 - rs15927400	**	0.47 ± 0.33	3	-
BMWmin	9	62	57 - 69	rs15977388 - rs15978241	*	0.42 ± 0.19	4	-
BMWmaj	9	66	59 - 82	rs14677393 - RBL2391	*	0.65 ± 0.07	2	-
BMP	9	66	61 - 70	rs14677393 - RBL2391	***, $	0.55 ± 0.24	3	-
AFP	10	59.8	56 - 68	rs14009177 - MCW0035	*	0.60 ± 0.17	2	-
AFW	10	62.8	56 - 75	rs14009888 - rs14010538	*	0.65 ± 0.02	2	-
*T*_b_	11	36	31 - 44	rs14025158 - rs15617411	**	0.44 ± 0.11	4	-
Glucose	18	30.2	23 - 38	rs14110229 - ADL0184	***, $	0.52 ± 0.19	4	-
ShD	19	18	0 - 22	rs14116183 - rs15837334	***, $	0.54 ± 0.16	3	-
BMW min	19	41	35 - 49	rs14120685 - RBL1230	*	0.47 ± 0.01	2	-
AFW	19	52	48 - 53	rs15855444 - rs14124107	***, $	0.52 ± 0.27	3	[32]
AFP	19	52	47 - 53	rs15855444 - rs14124107	***, $	0.52 ± 0.25	3	[32]
BW5	20	3.3	1 - 6	LEI0080 - rs14268358	*	0.60 ± 0.18	3	-
BW7	20	3.3	1 - 6	LEI0080 - rs14268358	*	0.60 ± 0.12	3	-
AFW	20	51.3	29 - 52	rs16175432 - rs14280613	*	0.60 ± 0.09	2	-
AFP	20	51.3	28 - 52	rs16175432 - rs14280613	*	0.59 ± 0.09	2	-
NEFA	21	34	31 - 36	RBL2361 - rs15184064	*	0.54 ± 0.19	4	-
AFW	26	45	18 - 46	rs16204669 - ADL0285	*	0.45 ± 0.12	3	-
ShD	27	28	19 - 36	RBL2860 - rs14303776	*	0.46 ± 0.32	3	-
ShL	27	32	18 - 40	rs14303776 - RBL4014	*	0.51 ± 0.22	3	-
ThP	27	48	33 - 53	ADL0376 - RBL10518	*	0.60 ± 0.18	3	-
AFW	27	51	47 - 53	ADL0376 - RBL10518	*	0.55 ± 0.25	3	-
AFP	27	51	47 - 53	ADL0376 - RBL10518	**	0.50 ± 0.26	4	[33]
ShL	28	41	28 - 51	MCW0227 - rs13726077	*	0.59 ± 0.19	2	-
BMP	28	51	47 - 51	rs14307413 - rs16212250	*	0.45 ± 0.25	3	-
BMW min	Z	88.9	86 - 99	RBL3035 - rs16110306	***, $	0.59 ± 0.32	4	-

Traits are described in Table 1; *GGA Gallus gallus* chromosome, *Loc* location, *CI* confidence interval, *SL* significance level with * at 5%, ** at 1%, *** at 0.1% chromosome-wide and $ at 5% genome-wide, *QTL effect* substitution effect expressed in residual standard deviation. *HS* number of heterozygous sires out of 5. *Ref* publications describing similar QTL affecting the same trait and presenting a similar genomic location.

QTL that affected growth, body composition and metabolic traits have been extensively studied in the chicken [6] and some of the QTL identified in this study had been previously described: AFW on GGA19 [32], AFP on GGA19 [32] and GGA27 [33], BMWmaj and BMWmin on GGA7 [31], BW5 on GGA3 [28] and GGA4 [30], BW7 on GGA4 [29] and IGF-I on GGA1 [27,29]. However, 38 of the 47 QTL identified here have not been described before. Conversely, some QTL that control glucose or NEFA that were previously identified in another chicken population were not observed here [34].

### Multiple-QTL analyses

Multiple-QTL analyses were performed to examine the potential presence of two QTL that segregate on the same chromosome and control the same trait. The results of multiple-QTL analyses are summarized in Table 5. The test of one-against-two QTL (H1 *vs.* H2) was first performed. Four pairs of QTL were identified and for each pair, the QTL previously detected under the single-QTL analysis was confirmed and a second QTL was identified. The new QTL on GGA3 at 96 cM that influenced BW5 had a high chromosome-wide significance (*P* < 0.01), while the remaining chromosome-wide QTL were significant at *P* < 0.05.

**Table 5:** Results of multiple QTL analysis

**Trait**	**GGA**	**QTL1 Loc (cM)**	**QTL1 nearest markers**	**QTL2 Loc (cM)**	**QTL2 nearest markers**	**H0 *vs *H2 SL**	**H1 *vs *H2 SL**	**QTL1 effect**	**HS**	**QTL2 effect**	**HS**	**Ref**
BMP	3	39	rs16225707- rs14316721	249	rs15452125- rs14410153	*		0.59 ± 0.30	2	0.75 ± 0.47	2	[29]
BW3	3	96	rs14334468- rs15316026	264	rs15457054- rs15459111	*		0.51 ± 0.16	3	0.54 ± 0.07	2	-
BW5	3	96	rs14334468- rs15316026	**252**	rs15452125- rs14410153		**	0.66 ± 0.13	2	0.52 ± 0.21	4	[28,30]
BW7	3	**96**	rs14334468- rs15316026	249	rs15452125- rs14410153		*	0.64 ± 0.20	2	0.65 ± 0.26	3	-
BW7	4	132	rs15595474- RBL4469	**150**	MCW0240- rs14488074		*	0.57 ± 0.19	5	0.72 ± 0.41	4	[29]
BMW maj	5	122	rs15731150- rs15733056	130	rs15733400- rs14551368	*		0.93 ± 0.12	2	0.60 ± 0.27	5	[35]
BMP	5	122	rs15731150- rs15733056	130	rs15733400- rs14551368	*		0.71 ± 0.40	3	0.62 ± 0.24	5	-
ThW	8	**10**	rs14635367- rs15900903	59	rs14648254- rs15925157		*	0.62 ± 0.15	2	0.49 ± 0.15	3	-
BW3	9	67	rs15979233- RBL4169	76	rs15981733- rs13735709	*		0.72 ± 0.43	4	0.69 ± 0.40	4	-
BMW maj	12	29	rs13610024- rs14036782	50	rs14981507- rs14043099	*		0.57 ± 0.20	4	0.65 ± 0.18	3	-
BMP	12	29	rs13610024- rs14036782	50	rs14981507- rs14043099	*		0.62 ± 0.21	4	0.56 ± 0.21	4	-
BW3	18	14	rs15813867- rs15037317	37	rs15826197- rs14112762	*		0.48 ± 0.15	4	0.65 ± 0.30	2	-

Traits are described in Table 1; *GGA Gallus gallus* chromosome, *Loc* location, *SL* significance level with * at 5% and ** at 1% chromosome-wide, *QTL* locations in bold, *QTLs* previously detected in single-QTL analysis, *QTL effect* substitution effect expressed in phenotypic standard deviation. *HS* number of heterozygous sires out of 5. *Ref* publications describing similar QTL affecting the same trait and presenting a similar genomic location.

The test of none-against-two QTL (H0 *vs.* H2) was then examined to test the possibility that two linked QTL located in a short interval might not be detected because of their antagonist effects. Eight new pairs of QTL were identified (*P* < 0.05) using this test.

Together, these two two-QTL analyses (H0 *vs.* H2 and H1 *vs.* H2 hypotheses), identified 20 additional QTL. Their effects ranged from 0.48 (BW3 on GGA18) to 0.93 (BMWmaj on GGA5) standard deviations, with a mean of 0.63 standard deviations. When a QTL was identified at a same location using H1 and H2 hypotheses, their effects were quite similar. Considering only the QTL detected under the H0 *vs.* H2 hypothesis, the average distance between the two QTL was 59 cM and ranged from 8 to 210 cM. Surprisingly, the two QTL that affected BMP and BW3 on GGA3 were very distant (210 cM and 168 cM, respectively). This may be because estimating two QTL effects together is more powerful and leads to a more precise QTL location [36]. However, excluding these two pairs of QTL, the average distance between the two QTL was 15 cM, consistent with the hypothesis of two closely located QTL with antagonist effects. Two of the QTL detected under the H0 *vs.* H2 hypothesis had previously been described in other populations, supporting the validity of the results for BMWmaj on GGA5 [35] and BMP on GGA3 [29].

When determining the threshold under the H1 *vs.* H2 hypothesis, one QTL was simulated using the location and effect previously identified under the single-QTL analysis. This is quite a conservative test, because if the location and effect were false (i.e. if a ghost QTL was detected under H1), the H2 hypothesis would likely be rejected. However, the second QTL affecting BW5 identified on GGA3 under this hypothesis was highly significant (*P* < 0.01), and has been previously described [28,30]. The QTL affecting BW7 and located on GGA4 has also been previously described [29]. Identification of highly significant QTL that have been previously described in other populations indicates that the results are robust.

### Candidate genes analyses

Many QTL were identified in this study, with intervals that contain for most of them a few dozen genes. From among these positional candidate genes, functional candidate genes were identified using the AnnotQTL web tool [37] for QTL regions that were significant at the genome level (*P* < 0.05). For BMWmin on GGA2 and *T*_b_ on GGA5, none of the described genes had a function that could be linked to the trait. However, many genes are not yet annotated or currently have a functional annotation that is difficult to link to the studied traits. Conversely, on GGA1, a very strong QTL that affects plasma IGF-I level is co-located with the gene that codes for the IGF-I protein. This strongly suggests that a mutation involved in IGF-I expression, putatively in the promoter of the IGF-I gene, could be responsible for the regulation of plasma IGF-I levels in the chicken. This mutation appears to be highly frequent in the population, since four of the five tested males were heterozygous for this QTL. Although the fat and lean chicken lines differ for circulating IGF-I levels at 9 weeks of age (FL > LL, [38]), the mutation does not appear to be specific to one of the founder lines, since allele origins were equally shared between the two lines. A similar QTL was previously suggested in an F_2_ population that resulted from the cross of chickens with high and low growth rates [34]. These lines have no common genetic background with the FL and LL lines. Up to 11 QTL for IGF-I plasma level have been described in different mouse strains ([39] and references therein) but the most significant QTL has been identified on mouse chromosome 10, where the *IGF1* gene is located. In mice, circulating IGF-I levels are closely correlated with IGF-I mRNA expression in the liver [40]. However, an eQTL search conducted on liver samples collected from 282 F_2_ mice failed to detect IGF-I cis-eQTL and thus did not support the hypothesis that a mutation in the regulatory regions of *IGF-I* could be responsible for the QTL that influences the level of circulating IGF-I [39]. Since liver samples were not collected from the FL × LL F_2_ population, this hypothesis could not be tested in the present study.

On GGA9, a QTL that affects BMP was identified. The gene *GHSR* (*growth hormone secretagogue receptor*), previously described as associated with chicken growth, specifically breast muscle weight [41], is located in this region. For glucose plasma concentration, a QTL was identified on GGA18. Two genes in this QTL interval could be interesting functional candidates: *DCXR* (*dicarbonyl/L-xylulose reductase*) and *ASPSCR1* (*alveolar soft part sarcoma chromosome region, candidate 1*). When overexpressed in transgenic mice, *DCXR* has been described as affecting blood level glucose [42]. ASPSCR1 interacts with glucose transporter type 4 (GLUT4), but no effect on glucose plasma concentration has been reported.

On GGA19, a highly significant novel QTL that affects both AFP and AFW was identified. This region contains the *ACACA* gene (*acetyl-CoA carboxylase A*), which is involved in fatty acid synthesis. A study of an SNP located in this gene has been found to be associated with fatness traits in chickens [32], enhancing the candidate status of this gene. A closer look at these genes might identify putative mutations that could be used for marker-assisted selection in chickens. On this same chromosome, but at a different location, another QTL affected ShD. A possible candidate gene for this QTL is *ChREBP* (*carbohydrate-responsive element-binding protein*), which is known to affect the fiber type transformation in skeletal muscle [43]. Finally, a QTL that affects BMWmin was observed on GGAZ. For this region, *GAS1* (*growth arrest-specific 1*) could be a good functional candidate gene, since it is involved in the cell cycle and has been described as promoting myogenic differentiation [44]. While these genes appear to be good functional candidates, it is necessary to identify and validate mutations to confirm their link with the observed QTL.

## Conclusions

This study enabled us to identify 67 QTL, many of which had not been described before. This result may be explained by the population used and the higher marker density, which increased marker coverage of micro-chromosomes. Because of the lack of markers on most micro-chromosomes in previous studies, only a few QTL had been identified on GGA19, 23, 24, 26 and 28, and none on GGA21, 22 and 25 [6]. In contrast, we identified QTL on GGA21 (NEFA), GGA19 (AFW, AFP, BMWmin and ShD), GGA26 (AFW), and GGA28 (BMP and ShL). In addition to greater genome coverage, the use of a larger set of SNPs made it possible to carry out multiple-QTL analyses and thus to detect many new QTL. This comprehensive study highlights several candidate genes that affect growth and body composition traits in meat-type chickens but further studies are required to confirm their role. Epistatic effects should also be considered, since such interactions have already been described in chicken for growth and body composition traits [29,45-48].

## Competing interests

The authors declared that they have no competing interests.

## Authors’ contributions

OD and ELBD managed the project. OD carried out the QTL mapping analyses and drafted the manuscript. ELBD supervised the QTL design and contributed to statistical analyses. NB helped in the interpretation of the data. MD, JS and ELBD managed the animal production and phenotyping. GLM performed part of the QTL mapping analyses. OF helped in the programming and the interpretation of the data. FP and AB supervised the genotyping. SL was involved in the statistical analyses. PLR helped in the QTL mapping and the interpretation of the data. LC and JS contributed to the funding and design of the experiment. All authors read and approved the final manuscript.

## References

[B1] Arthur JA Albers GAA Muir WM Industrial perspective on problems and issues associated with poultry breeding Poultry Genetics, Breeding and Biotechnology 2003 Cambridge: CABI Publishing 1 12

[B2] Eisen EJ Selection experiment for body composition in mice and rats: a review Livest Prod Sci 1989 23 17 32 10.1016/0301-6226(89)90003-1

[B3] Leclercq B Whitehead CC Leanness in Domestic Birds: Genetic, Metabolic and Hormonal Aspects 1988 London: Butterworths

[B4] Geraert PA MacLeod MG Larbier M Leclercq B Nitrogen metabolism in genetically fat and lean chickens Poult Sci 1990 69 1911 1921 10.3382/ps.0691911 2087450

[B5] Leclercq B Blum JC Boyer JP Selecting broilers for low or high abdominal fat: initial observations Br Poult Sci 1980 21 107 113 10.1080/00071668008416644

[B6] Abasht B Dekkers JC Lamont SJ Review of quantitative trait loci identified in the chicken Poult Sci 2006 85 2079 2096 17135661 10.1093/ps/85.12.2079

[B7] Wong GK Liu B Wang J Zhang Y Yang X Zhang Z Meng Q Zhou J Li D Zhang J Ni P Li S Ran L Li H Zhang J Li R Li S Zheng H Lin W Li G Wang X Zhao W Li J Ye C Dai M Ruan J Zhou Y Li Y He X Zhang Y Wang J A genetic variation map for chicken with 2.8 million single-nucleotide polymorphisms Nature 2004 432 717 722 10.1038/nature03156 15592405 PMC2263125

[B8] Baéza E Le Bihan-Duval E Chicken lines divergent for low or high abdominal fat deposition: a relevant model to study the regulation of energy metabolism Animal 2013 7 965 973 10.1017/S1751731113000153 23433003

[B9] Leclercq B Simon J Karmann H Glucagon-Insulin balance in genetically lean or fat chickens Diabete Metab 1988 14 641 645 3069508

[B10] Abasht B Pitel F Lagarrigue S Le Bihan-Duval E Le Roy P Demeure O Vignoles F Simon J Cogburn L Aggrey S Vignal A Douaire M Fatness QTL on chicken chromosome 5 and interaction with sex Genet Sel Evol 2006 38 297 311 10.1186/1297-9686-38-3-297 16635451 PMC2733900

[B11] Lagarrigue S Pitel F Carre W Abasht B Le Roy P Neau A Amigues Y Sourdioux M Simon J Cogburn L Aggrey S Leclercq B Vignal A Douaire M Mapping quantitative trait loci affecting fatness and breast muscle weight in meat-type chicken lines divergently selected on abdominal fatness Genet Sel Evol 2006 38 85 97 10.1186/1297-9686-38-1-85 16451793 PMC2689300

[B12] Elsen JM Mangin B Goffinet B Boichard D Le Roy P Alternative models for QTL detection in livestock. I. General introduction Genet Sel Evol 1999 31 213 224 10.1186/1297-9686-31-3-213

[B13] Filangi O Moreno C Gilbert H Legarra A Le Roy P Elsen J QTLMap, a software for QTL detection in outbred populations Proceedings of the 9th World Congress on Genetics Applied to Livestock Production: 1–6 August 2010 2010 Leipzig ID787 http://www.kongressband.de/wcgalp2010/assets/pdf/0787.pdf

[B14] Enright WJ Chapin LT Moseley WM Zinn SA Kamdar MB Krabill LF Tucker HA Effects of infusions of various doses of bovine growth hormone-releasing factor on blood hormones and metabolites in lactating Holstein cows J Endocrinol 1989 122 671 679 10.1677/joe.0.1220671 2509616

[B15] Demeure O Lecerf F MarkerSet: a marker selection tool based on markers location and informativity in experimental designs BMC Res Notes 2008 1 9 10.1186/1756-0500-1-9 18710478 PMC2525642

[B16] de Givry S Palhiere I Vitezica Z Schiex T Mendelian error detection in complex pedigree using weighted constraint satisfaction techniques Constraints 2008 13 130 154 10.1007/s10601-007-9029-5

[B17] R Development Core Team R Foundation for Statistical Computing R: A language and environment for statistical computing 2005 Vienna: R Foundation for Statistical Computing http://www.R-project.org

[B18] Groeneveld E Kovac M Mielenz N VCE User’s Guide and Reference Manual Version 6.0 2010 http://vce.tzv.fal.de/software/documentation/manual

[B19] Groenen MA Wahlberg P Foglio M Cheng HH Megens HJ Crooijmans RP Besnier F Lathrop M Muir WM Wong GK Gut I Andersson L A high-density SNP-based linkage map of the chicken genome reveals sequence features correlated with recombination rate Genome Res 2009 19 510 519 19088305 10.1101/gr.086538.108 PMC2661806

[B20] Elsen JM Filangi O Gilbert H Le Roy P Moreno C A fast algorithm for estimating transmission probabilities in QTL detection designs with dense maps Genet Sel Evol 2009 41 50 10.1186/1297-9686-41-50 19919698 PMC2793243

[B21] Chapuis G Filangi O Elsen JM Lavenier D Le Roy P GPU accelerated QTL detection J Comput Biol 2013 in press

[B22] Gilbert H Le Roy P Methods for the detection of multiple linked QTL applied to a mixture of full and half sib families Genet Sel Evol 2007 39 139 158 10.1186/1297-9686-39-2-139 17306198 PMC2682834

[B23] Ott J Analysis of Human Genetic Linkage 1991 3 London: John Hopkins University Press

[B24] Harrel FE Davis CE A new distribution-free quantile estimator Biometrika 1982 69 635 640 10.1093/biomet/69.3.635

[B25] Qanbari S Hansen M Weigend S Preisinger R Simianer H Linkage disequilibrium reveals different demographic history in egg laying chickens BMC Genet 2010 11 103 21078133 10.1186/1471-2156-11-103 PMC3001694

[B26] Park HB Jacobsson L Wahlberg P Siegel PB Andersson L QTL analysis of body composition and metabolic traits in an intercross between chicken lines divergently selected for growth Physiol Genomics 2006 25 216 223 10.1152/physiolgenomics.00113.2005 16390876

[B27] Zhou H Evock-Clover CM McMurtry JP Ashwell CM Lamont SJ Genome-wide linkage analysis to identify chromosomal regions affecting phenotypic traits in the chicken. IV. Metabolic traits Poult Sci 2007 86 267 276 17234839 10.1093/ps/86.2.267

[B28] Zhang S Li H Shi H Single marker and haplotype analysis of the chicken apolipoprotein B gene T123G and D9500D9- polymorphism reveals association with body growth and obesity Poult Sci 2006 85 178 184 16523611 10.1093/ps/85.2.178

[B29] Ankra-Badu GA Shriner D Le Bihan-Duval E Mignon-Grasteau S Pitel F Beaumont C Duclos MJ Simon J Porter TE Vignal A Cogburn LA Allison DB Yi N Aggrey SE Mapping main, epistatic and sex-specific QTL for body composition in a chicken population divergently selected for low or high growth rate BMC Genomics 2010 11 107 10.1186/1471-2164-11-107 20149241 PMC2830984

[B30] Ambo M Moura AS Ledur MC Pinto LF Baron EE Ruy DC Nones K Campos RL Boschiero C Burt DW Coutinho LL Quantitative trait loci for performance traits in a broiler x layer cross Anim Genet 2009 40 200 208 10.1111/j.1365-2052.2008.01824.x 19170675

[B31] Atzmon G Blum S Feldman M Cahaner A Lavi U Hillel J QTLs detected in a multigenerational resource chicken population J Hered 2008 99 528 538 10.1093/jhered/esn030 18492652

[B32] Tian J Wang S Wang Q Leng L Hu X Li H A single nucleotide polymorphism of chicken acetyl-CoA carboxylase A gene associated with fatness traits Anim Biotechnol 2010 21 42 50 20024786 10.1080/10495390903347009

[B33] Abasht B Lamont SJ Genome-wide association analysis reveals cryptic alleles as an important factor in heterosis for fatness in chicken F2 population Anim Genet 2007 38 491 498 10.1111/j.1365-2052.2007.01642.x 17894563

[B34] Nadaf J Pitel F Gilbert H Duclos MJ Vignoles F Beaumont C Vignal A Porter TE Cogburn LA Aggrey SE Simon J Le Bihan-Duval E QTL for several metabolic traits map to loci controlling growth and body composition in an F2 intercross between high- and low-growth chicken lines Physiol Genomics 2009 38 241 249 10.1152/physiolgenomics.90384.2008 19531576

[B35] Le Mignon G Pitel F Gilbert H Le Bihan-Duval E Vignoles F Demeure O Lagarrigue S Simon J Cogburn LA Aggrey SE Douaire M Le Roy P A comprehensive analysis of QTL for abdominal fat and breast muscle weights on chicken chromosome 5 using a multivariate approach Anim Genet 2009 40 157 164 10.1111/j.1365-2052.2008.01817.x 19243366

[B36] Blangero J Almasy L Multipoint oligogenic linkage analysis of quantitative traits Genet Epidemiol 1997 14 959 964 10.1002/(SICI)1098-2272(1997)14:6<959::AID-GEPI66>3.0.CO;2-K 9433607

[B37] Lecerf F Bretaudeau A Sallou O Desert C Blum Y Lagarrigue S Demeure O AnnotQTL: a new tool to gather functional and comparative information on a genomic region Nucleic Acids Res 2011 39 W328 W333 10.1093/nar/gkr361 21596783 PMC3125768

[B38] Beccavin C Chevalier B Simon J Duclos MJ Circulating insulin-like growth factors (IGF-I and -II) and IGF binding proteins in divergently selected fat or lean chickens: effect of prolonged fasting Growth Horm IGF Res 1999 9 187 194 10.1054/ghir.1999.0109 10502455

[B39] Leduc MS Hageman RS Meng Q Verdugo RA Tsaih SW Churchill GA Paigen B Yuan R Identification of genetic determinants of IGF-1 levels and longevity among mouse inbred strains Aging Cell 2010 9 823 836 10.1111/j.1474-9726.2010.00612.x 20735370 PMC3025299

[B40] Adamo ML Ma X Ackert-Bicknell CL Donahue LR Beamer WG Rosen CJ Genetic increase in serum insulin-like growth factor-I (IGF-I) in C3H/HeJ compared with C57BL/6J mice is associated with increased transcription from the IGF-I exon 2 promoter Endocrinology 2006 147 2944 2955 10.1210/en.2005-0742 16527837

[B41] Fang M Nie Q Luo C Zhang D Zhang X Associations of GHSR gene polymorphisms with chicken growth and carcass traits Mol Biol Rep 2010 37 423 428 10.1007/s11033-009-9556-9 19437137

[B42] Sudo T Ishii A Asami J Uematsu Y Saitoh M Nakamura A Tada N Ohnuki T Komurasaki T Nakagawa J Transgenic mice over-expressing dicarbonyl/L-xylulose reductase gene crossed with KK-Ay diabetic model mice: an animal model for the metabolism of renal carbonyl compounds Exp Anim 2005 54 385 394 10.1538/expanim.54.385 16365515

[B43] Hanke N Scheibe RJ Manukjan G Ewers D Umeda PK Chang KC Kubis HP Gros G Meissner JD Gene regulation mediating fiber-type transformation in skeletal muscle cells is partly glucose- and ChREBP-dependent Biochim Biophys Acta 1813 2011 377 389 10.1016/j.bbamcr.2010.12.021 21215280

[B44] Leem YE Han JW Lee HJ Ha HL Kwon YL Ho SM Kim BG Tran P Bae GU Kang JS Gas1 cooperates with Cdo and promotes myogenic differentiation via activation of p38MAPK Cell Signal 2011 23 2021 2029 10.1016/j.cellsig.2011.07.016 21820049

[B45] Carlborg O Hocking PM Burt DW Haley CS Simultaneous mapping of epistatic QTL in chickens reveals clusters of QTL pairs with similar genetic effects on growth Genet Res 2004 83 197 209 10.1017/S0016672304006779 15462413

[B46] Carlborg O Kerje S Schütz K Jacobsson L Jensen P Andersson L A global search reveals epistatic interaction between QTL for early growth in the chicken Genome Res 2003 13 413 421 10.1101/gr.528003 12618372 PMC430275

[B47] Le Rouzic A Alvarez-Castro JM Carlborg O Dissection of the genetic architecture of body weight in chicken reveals the impact of epistasis on domestication traits Genetics 2008 179 1591 1599 10.1534/genetics.108.089300 18622035 PMC2475757

[B48] Wahlberg P Carlborg O Foglio M Tordoir X Syvänen AC Lathrop M Gut IG Siegel PB Andersson L Genetic analysis of an F(2) intercross between two chicken lines divergently selected for body-weight BMC Genomics 2009 10 248 10.1186/1471-2164-10-248 19473501 PMC2695486

